# Competent and deficient provision of childbirth services: a descriptive observational study assessing the quality of intrapartum care in two provinces of the Democratic Republic of the Congo

**DOI:** 10.1186/s12913-022-07737-5

**Published:** 2022-04-25

**Authors:** Stephan Brenner, Supriya Madhavan, Céline Kanionga Nseya, Claude Sese, Günther Fink, Gil Shapira

**Affiliations:** 1 Heidelberg Institute of Global Health, University Heidelberg, Im Neuenheimer Feld 130.3, 69120 Heidelberg, Germany; 2 Global Financing Facility and The World Bank, Washington, DC, USA; 3 Institut Supérieur des Techniques Médicales, Kinshasa, Democratic Republic of the Congo; 4 Bureau Appui Technique, Division Provinciale de la Santé, Kinshasa, Democratic Republic of the Congo; 5 Programme de Développement du Système de Santé (PDSS), Ministry of Public Health, Kinshasa, Democratic Republic of the Congo; 6 Swiss Tropical and Public Health Institute, Basel, Switzerland; 7 University of Basel, Basel, Switzerland; 8 Development Research Group (DECRG), The World Bank, Washington, DC, USA

**Keywords:** Quality of care, Intrapartum care, Maternal care, Democratic Republic of the Congo

## Abstract

**Background:**

A majority of women in the Democratic Republic of the Congo (DRC) give birth in a health facility, but maternal and newborn mortality remains high. In rural areas, the quality of facility-based delivery care is often low. This study examines clinical quality of intrapartum care in two provinces of the DRC.

**Methods:**

We observed process and input elements of delivery care provision at 29 facilities in Kwilu and Kwango provinces. Distinguishing non-performance attributable to provider behavior vs. input constraints, we compared both providers’ adherence to clinical standards (“competent care”) and non-adherence to processes for which required inputs were available (“deficient care”).

**Results:**

Observing a total of 69 deliveries, care was most competent for partograph use (75% cases) and hemorrhage prevention (73%), but least for postpartum monitoring (4%). Competent care was significantly associated with higher case volumes (*p* = ·03), skilled birth attendance (*p* = ·05), and nulliparous women (*p* = ·02). Care was most deficient for infection prevention (62%) and timely care (49%) and associated with cases observed at hospitals and lower delivery volume.

**Conclusions:**

Low quality was commonly not a result of missing equipment or supplies but related to providers’ non-adherence to standard protocols. Low case volumes and the absence of skilled attendants seemed to be main factors for sub-standard quality care. Birth assistance during labor stage 2 was the only intrapartum stage heavily affected by the unavailability of essential equipment. Future interventions should strengthen links between birth attendants’ practice to clinical protocols.

## Background

A majority of pregnancy-related maternal deaths, a third of newborn deaths, and half of all still births in Sub-Saharan Africa occur during the intrapartum period or the 24 h following birth [1]. Hemorrhage, hypertension, and sepsis represent leading direct causes of maternal mortality, perinatal asphyxia and birth-related infections are main causes of newborn deaths [1]. Limitations in timely care provision further contribute to intrapartum mortality, especially in instances when health facilities lack essential supplies or less qualified care providers [2].

The Democratic Republic of the Congo (DRC) is the largest country in the Central African region. As the largest country by area (2.3 million km^2^) as well as the third most populous country in Sub-Saharan Africa (92 million people), population density is overall low (41 inhabitants/square kilometer) [3]. Especially outside major cities, political instability, corruption, and underinvestment in transport infrastructure has limited the development of a strong healthcare system that guarantees reliable access to primary care for people living in more remote provinces [4]. As a result, quality of maternal and newborn health services has be low, especially in rural areas [5].

The DRC ranks among the five countries with highest proportions of maternal and neonatal deaths worldwide [6, 7]. In 2017, the DRC reported 846 maternal deaths per 100,000 livebirths and t 28 newborn deaths per 1000 livebirths [8], although a rather high proportion of births (about 80%) occur in health facilities [8]. However, skilled birth attendance varies greatly (65–98% of births) with lower coverage in poorer and rural areas [8]. While, only about 8% of maternal care facilities lack essential childbirth equipment and supplies [9], about 90% of birth attendants had never undergone any specific trainings in preventing maternal and newborn mortality, such as active third stage labor management, essential newborn care, or emergency obstetric care [9].

Based on this evidence, it appears that pregnant women are largely able to access childbirth care at sufficiently equipped facilities, even in rural areas of the DRC. Therefore, it is plausible that the discouraging maternal and newborn mortality outcomes are driven by limited quality of care processes rather than access to care. However, little is understood about maternal care provision in the rural provinces. In this observational study, we assess the content of care provided to women giving birth in rural health facilities in the DRC and describe the observed quality of care in relation to facilities’ capacity to provide essential obstetric care.

## Methods

### Study Setting

In the DRC, health facilities are organized in health zones (*Zones de Santé*), each zone comprising at least one hospital (*Hôpital Général de Référence*) and several health centers (*Centres de Santé*). Together, these health facilities are responsible for implementing the country’s primary health care strategy, including standards related to maternal, newborn and child health.

This study reports observed clinical practices of birth attendants in two rural provinces, Kwilu and Kwango, with largely comparable maternal care statistics. In 2018, maternal mortality in Kwilu and Kwango was with 312 and 442 deaths per 100,000 live births, respectively, below national average [10]. Further, a vast majority of health facilities in both provinces offered essential obstetric care (96% in Kwilu, 95% in Kwango) [9], while hardly any of these facilities had the capacity to provide emergency obstetric care [11]. The two provinces however differed in the proportion of births attended by a skilled birth attendant, with 93% of births in Kwilu much higher compared to Kwango with 61% [8].

### Study design and sampling

As outlined above, the purpose of this observational study was to assess the care provided to women giving birth in rural health facilities as well as whether gaps in clinical protocol adherence are driven by the availability of medical supplies. To do so, we combined two existing data streams collected by the *Projet d’Appui des Services de Santé* (PDSS), a World Bank Funded project that aims at improving maternal and child healthcare provision [12]. Although the project supports health zones in 11 provinces, five health zones in two provinces were purposively selected for this study. Three of the selected health zones (Gungu, Mosango, Pay Kongila) are in Kwilu and in two health zones (Boko, Kimbao) are in Kwango. Each of these five health zones comprises of one referral hospital and between 1*6* and 26 health centers. All referral hospitals were included in this study, as well health centers randomly selected for a wider PDSS evaluation. Ten health centers were selected in Gungu, six in Kimbao, and five in each of the other zones. The study sample therefore included a total of 36 facilities.

Direct clinical observations of intrapartum care delivery were conducted at these 36 facilities. For this study, all non-complicated childbirth cases (i.e. normal vaginal births) presenting to each of these facilities during a five-day period were observed. Based on PDSS program data on childbirth frequencies in these facilities from 2017 (the year preceding the case observations) it was estimated that 5 days of observation at each facility would on average yield 2.6 deliveries in health centers located in both Kwango provinces. Given the logistic challenges for having research teams safely move between study facilities, a five-day observation stay at each facility was therefore the most feasible field strategy. In total, 69 case observations could be observed at 29 facilities (i.e. at seven health centers no births could be observed during the five-day stay given the low case volume).

### Study data

Content of care data was directly observed between August and September 2018 and contained information on the following intrapartum aspects: labor room set-up, patient history and physical assessment, labor monitoring, birth assistance, immediate newborn care, placenta management, and immediate postpartum monitoring. Information on birth attendants’ professional qualifications and patients’ parity status was also recorded. Case observation started once the laboring woman was admitted to the labor ward and ended 2 hours after birth. Observed information was recorded in a checklist. Checklist content reflected clinical standards taken from national treatment protocols for essential intrapartum and immediate newborn care [13]. Prior to conducting an observation, informed consent was obtained from both the involved birth attendants and the patient.

Case observations were conducted by trained data collectors with a background in midwifery. Prior to data collection, data collectors participated in a 4-day training workshop, followed by a 2-day pilot to ensure observed activities were consistently observed and recorded across data collectors. During actual field data collection activities, data collectors were assigned to trained peer supervisors who ensured that recorded data met the completeness and quality standards.

Further, all 36 PDSS facilities enrolled under the financing scheme report quarterly inventory data, including information on their’ capacity to provide childbirth services. For intrapartum care, this data included information on key input elements, such as the availability of essential equipment and supplies. To ensure timely overlap with the observed case data, we used quarterly facility data reported for the quarter July–September 2018.

### Definition of study variables

#### Conceptual approach to intrapartum care quality measures

With a focus on technical quality only, available data points measured providers’ adherence to clinical intrapartum standards [14]. We assigned observed process measures to one of five clinical intrapartum stages: “labor monitoring”, “assistance in childbirth”, “assistance in delivery of placenta”, “immediate postpartum monitoring”, and “cross-cutting intrapartum care”. For each intrapartum stage, we then identified those indicators directly aligned with the concepts of “competent care” (i.e. evidence-based, effective care) and “capable systems “(i.e. environments that ensure patient safety, prevention and detection of negative health, continuity of care, and timely action) suggested in the Lancet Global Health Commission’s health systems assessment framework [15].

#### Definition of competent care

Our process selecting competent care indicators followed the rationale to focus on intrapartum care processes that could be directly linked to those clinical activities that prevent or control direct causes of maternal or newborn death (i.e. maternal and newborn sepsis, asphyxia, fetal distress, newborn distress, postpartum hemorrhage) and included a detailed review key documents related to intrapartum care provision at the community level [16–18], as well as by our previous work on routine intrapartum care assessment [19]. Resulting indicator definitions for each competent care domain are shown in Table 1 and include: “patient safety” (defined by infection prevention measures throughout case management), “early detection” (defined by the use of a partograph to identify signs of adverse labor progression during stage 1 labor), “timely access” (defined as accessibility to key birth equipment during stage 2 labor), “prevention” (defined as measures reducing the risk of postpartum hemorrhage during stage 3 labor), and “continuity of care” (defined as measures monitoring maternal and newborn well-being in the immediate postpartum). We identified two process indicators for each competent care domain, except for “early detection” with only one indicator.

**Table 1:** Outline of domains, indictors, definitions, and rationale applied to assessment of competent intrapartum care processes

Competent intrapartum care domains	Competent care indicators	Indicator definitions:	Rationale for indicator selection with respect to positive birth outcome:
**Patient safety** hygiene practices limiting healthcare-associated infections throughout intrapartum period.	“Sterile precautions during vaginal examination”	“Handwashing/hand disinfection and use of sterile gloves with each vaginal examination.” **Required input elements:** Handwashing facility (any of the following: tap, water in a bucket with fitted tap, pitcher located inside maternity), sterile gloves (at least 5 pairs for health centers, 10 pairs for hospitals)	Vaginal examinations conducted in a sterile manner to limit risk of endometrial infections.
	“Sterile precautions during assistance of childbirth”	“Handwashing/hand disinfection and use of sterile gloves during stage 2 labor and immediate newborn care.” **Required input elements:** Handwashing facility (any of the following: tap, water in a bucket with fitted tap, pitcher located inside maternity), sterile gloves (at least 5 pairs for health centers, 10 pairs for hospitals)	Birth assisted in a sterile manner to lower risk of endometrial and umbilical infections.
**Early detection** of adverse progression during stage 1 labor monitoring.	“Partograph use”	“Labor progression plotted on partograph in real time.” **Required input elements:** Partographs (at least 10 blank forms)	Routine monitoring of maternal and fetal well-being to detect abnormal labor progression during stage 1.
**Timely access** to key equipment to limit third delay during stage 2 birth assistance.	“Delivery equipment instantly accessible”	“Delivery instruments (e.g. sterile delivery kit) made directly accessible in delivery room prior to onset of stage 2 labor.” **Required input elements:** Delivery instruments (either: 2 sterile delivery kits, or: all following items: 4 blades, 4 clamps/ties, 2 needles & holders, 4 forceps)	Vaginal birth is assisted in an immediate manner, especially in case of fetal or maternal distress.
	“Aspirator device instantly accessible”	Aspirator device made directly accessible in delivery room prior to onset of stage 2 labor. **Required input elements:** Aspirator device (either functional manual or electric vacuum aspirator)	Obstructed airway is managed in an immediate manner, especially in case of newborn asphyxia.
**Prevention** of postpartum hemorrhage during stage 3 birth management.	“Use of uterotonic”	Administration of uterotonic in beginning of stage 3. **Required input elements:** Oxytocin: at least 1 oxytocin injection.	Pharmacological prevention of postpartum bleeding during stage 3.
	“Placenta completeness”	Once delivered, placenta is examined for completeness.	Postpartum bleeding as result of retained products is excluded.
**Continuity of care** during stage 4 immediate postpartum monitoring.	“Maternal monitoring”	Blood pressure, heart rate, uterus tone, and vaginal bleeding checked at least once during initial 2 h following childbirth.	Signs of postpartum bleeding continued to be monitored after childbirth.
	“Newborn monitoring”	Breathing effort, temperature, general responsiveness checked at least once during initial 2 h following childbirth.	Signs of neonatal distress continued to be monitored after childbirth.

#### Definition of deficient care

For clinical processes dependent on essential input elements, we further assessed “deficient care” defined as absent provider performance although the essential input element (e.g. equipment or supplies as outlined in Table 1) would have been available at the facility. For instance, and as outlined in Table 5, infection prevention was considered deficient if any of its two processes (handwashing, glove use) was not observed in a case although the facility had the respective inputs (water source, sterile gloves) available. Selection of essential input elements followed a review of international recommendations and standards [16, 20].

#### Additional variables

We assumed childbirth services provided at the hospital level to be of relative higher quality of care compared to health centers [21]. The variable “facility type” therefore classifies facilities as hospitals or health centers to reflect the stronger human and technical capacities (e.g. higher staff numbers including physicians or gynecologists, and more diagnostic and therapeutic options with respect to the management of pregnancy complications) accessible to hospitals.

We further assumed that a higher volume of childbirth cases would also result in higher quality of intrapartum care as provided care at high volume facilities would be linked to more frequent exposure to different cases [22]. The variable “delivery volume” represents a facility’s annual volume of assisted non-complicated delivery cases (data taken from PDSS routine data for the year 2017) classifying facilities as “higher” or “lower” volume based on a cut-off at 183 noncomplicated births per year (i.e. equivalent to one delivery every other day).

We also assumed that the number of skilled birth attendants (SBAs) at a facility will positively influence observed quality. The variable “presence of at least one SBA” indicates whether a SBA was present in the maternity during case observation, and if so, whether or not this SBA was directly or indirectly (i.e. closely supervising a non-skilled provider) in the care process. Our definition of SBAs is based on national provider qualification categories used in the DRC (i.e. midwives and nurse-midwives with at least 2 years undergraduate training, as well as gynecologists), assuming more competent care provided by skilled providers [13, 23].

Lastly, given the higher risk for negative birth outcomes for nulliparous women, we expected intrapartum care provided to these patients to more closely follow standard protocols [24]. The variable “client’s parity” groups cases into those involving a woman without previous childbirths (nulliparous) and those with one or more previous births (multiparous).

### Analysis

Our analysis primarily focused on the 29 facilities for which case data could be observed to understand the overall frequency of both competent and deficient care as well as their association with observed facility and case characteristics. For each case, measures of competent care were computed based on the observed information. To compute deficient care measures, observed case data was matched with the respective facility’s capacity assessment. We then computed the frequencies of both identified competent and identified deficient care processes by indicator and care domains. To explore associations of frequency distributions with respect to the facility and case level characteristics described above we used. Fisher’s exact tests All analysis were computed using Stata.

## Results

### Sample characteristics

#### Facility sample

Of the 36 facilities surveyed, we were able to observe at minimum one birth per five-day observation period in only 29 facilities (Table 2). The seven facilities without any observed delivery were all health centers, approximately evenly distributed across the five health zones, and fell into our category of lower delivery case volumes (data not shown). Delivery volumes for facilities with observed cases ranged between 7 and 323 (mean 176) deliveries in previous year for health centers and 73–207 (mean 148) for hospitals (data not shown). About 59% of facilities (three hospitals and 14 health centers) managed on average less than one case every other day in the previous year (Table 2).

**Table 2:** Facility and case sample sizes and sample distribution by key characteristics

			n	(%)
**Total facility sample**			**29**	**(100)**
**Key facility characteristics**	Province:	Kwilu	18	(62·1)
		Kwango	11	(37·9)
	Facility type:	Health center	24	(82·8)
		Hospital	5	(17·2)
	Delivery volume: ^a^	High	12	(41·4
		Low	17	(58·6)
**Total case sample**			**69**	**(100)**
**Key case characteristics**	Facility type:	Health center	55	(79·7)
		Hospital	14	(20·3)
	Delivery volume: ^a^	High	31	(44·9)
		Low	38	(55·1)
	Skilled birth attendant (SBA) present: ^b^	At least 1 SBA present in maternity at time of case observation	24	(34·8)
		No SBA present in maternity	45	(65·2)
	Client’s parity status:	Nulliparous	23	(33·3)
		Multiparous	46	(66·7)

^a^ cutoff defined at 183 delivery cases in previous calendar year (i.e. on average one case every other day)

^b^ “skilled” includes nurses, midwives or other maternal care providers with at least any of the following training qualifications: A0 = 2-year postgraduate training, A1 = 3-year undergraduate training, A2 = 2-year undergraduate training

#### Case sample

We were able to observe a total of 69 uncomplicated births across these 29 facilities. About 80% of cases were observed at health centers and about 45% at higher-volume facilities (Table 2). One third of cases involved a nulliparous woman and about 35% of cases had a skilled provider (i.e. a midwife, nurse or other maternal care provider with at least 2 years of undergraduate training) present in the maternity.

### Competent care and association with key characteristics

As shown in Table 3, the highest frequencies of competent intrapartum care provision was observed for the domains “early detection” (i.e. 75% of cases monitored by partograph) and “prevention” (73% of cases). The latter included the indicator assessing oxytocin administration during stage 3, which was with 91% of cases the most frequently observed process overall. Competent care for the domains “patient safety” and “timely access” was observed in only 20 and 17% of cases, respectively. Continuity of competent care during the immediate postpartum period was nearly absent and observed in only three cases (4%).

**Table 3:** Observed competent care by domains and related indicators

Competent care by domain	n	(%)	Competent care processes by indicator	n	(%)
Patient safety	14	(20·3)	“Sterile precautions during vaginal examination”	15	(21·8)
			“Sterile precautions during assisted childbirth”	31	(44·9)
Early detection	52	(75·4)	“Partograph use”	52	(75·4)
Timely access	12	(17·4)	“Delivery equipment instantly accessible”	42	(60·9)
			“Aspirator device instantly accessible”	17	(26·6)
Prevention	50	(72·5)	“Use of uterotonic”	63	(91·3))
			“Placenta completeness”	52	(75·4)
Continuity of care	3	(4·4)	“Maternal monitoring”	8	(11·2)
			“Newborn monitoring”	5	(7·3)

Total *n* = 69 cases

As shown in Table 4, statistically significant associations between observed competent care and key facility or case characteristics existed between delivery volume and “timely access”, with a positive association between competent care provision and cases managed at higher-volume facilities. Significant positive associations also existed between SBA involvement and “prevention” as well as between cases involving a nulliparous woman and both “early detection” and “timely access”.

**Table 4:** Associations of key characteristics with observed competent care by domain

Key characteristics		Patient safety			Early detection			Timely access			Prevention			Continuity		
		n	(%)	***p***	n	(%)	***p***	n	(%)	***p***	n	(%)	***p***	n	(%)	***p***
Facility type:	Health center	13	(23·6)		11	(78·6)		10	(18·2)		38	(69·1)		2	(3·6)	
	Hospital	1	(7·1)	*·27*	41	(74·6)	*·99*	2	(14·3)	*·99*	12	(85·7)	*·32*	1	(7·1)	*·50*
Delivery volume: ^a^	High	8	(25·8)		25	(80·7)		9	(29·0)		25	(80·7)		1	(3·2)	
	Low	6	(15·8)	*·37*	27	(71·1)	*·41*	3	(7·9)	*·****03***	25	(65·8)	*·19*	2	(5·3)	*·99*
Direct/indirect involvement of skilled attendant: ^b^	Yes	4	(26·7)		7	(77·8)		4	(40·0)		10	(100)		0	(0)	
	No	10	(18·5)	*·49*	45	(75·0)	*·99*	8	(13·6)	*·****06***	40	(67·8)	***·05***	3	(5·0)	*·99*
Client’s parity status:	Nulliparous	5	(21·7)		21	(91·3)		8	(34·8)		17	(73·9)		1	(4·4)	
	Multiparous	9	(19·6)	*·99*	31	(67·4)	*·****04***	4	(8·7)	*·****02***	33	(71·7)	*·99*	2	(4·4)	*·99*

Total *n* = 69 cases, numbers and frequencies shown represent cases with observed care within in each categorical expression of the reported characteristic

*p*-values based on Fisher’s exact test

^a^ cutoff defined at 183 delivery cases in previous calendar year (i.e. on average one case every other day)

^b^ “skilled” includes nurses, midwives or other maternal care providers with any of the following training qualifications: A0 = 2-year postgraduate training, A1 = 3-year undergraduate training, A2 = 2-year undergraduate training; direct involvement = skilled provide directly performs observed care domain, indirect involvement = skilled provider supervises non-skilled provider in executing the observed care domain

### Deficient care

Table 5 shows the frequencies of cases with non-adherence to selected processes for which essential key inputs were actually available at the respective facility. For “patient safety” competent care was deficient in 62% of cases, largely due to ineffective infection precautions taken during vaginal examinations. “Timely access” was deficient in 49% of cases due to aspirator devices not made instantly accessible during stage 2 labor. “Early detection” was deficient in 22% and “prevention” in 7% of cases.

**Table 5:** Observed deficient care by domains and related indicator combinations

Deficient care by domain	n	(%)	Deficient care indicator combinations	n	(%)
Patient safety	43	(62·3)	“Water source and sterile gloves available” PLUS “No sterile precautions during vaginal examination”	42	(60·9)
			“Water source and sterile gloves available” PLUS “No sterile precautions during assisted childbirth”	29	(42·0)
Early detection	15	(21·7)	“Partographs available” PLUS “No partograph use”	15	(21·7)
Timely access	34	(49·3)	“Delivery equipment available “PLUS “No instant access to delivery equipment”	16	(23·2)
			“Aspirator device available “PLUS “No instant access to aspirator device”	34	(49·3)
Prevention	5	(7·3)	“Oxytocin available” PLUS “No uterotonic use”	5	(7·3)

Total *n* = 69 cases

As shown in Table 6, statistically significant relationships between deficient care and key characteristics existed for facility type with a positive association between “deficient patient safety” and cases managed at hospitals. Significant positive associations also existed between a lower delivery volume and “deficient prevention”, which was largely due to the omission of oxytocin administration in cases at lower volume facilities.

**Table 6:** Associations of key characteristics with observed deficient care by domain

Key characteristics		Patient safety			Early Detection			Timely Access			Prevention		
		n	(%)	***p***	n	(%)	***p***	n	(%)	***p***	n	(%)	***p***
Facility type:	Health center	30	(54·6)		12	(21·8)		27	(49·1)		5	(9·1)	
	Hospital	13	(92·9)	*·****01***	3	(21·4)	*·99*	7	(50·0)	*·99*	0	(0)	*·58*
Delivery volume: ^a^	High	21	(67·7)		4	(12·9)		18	(58·1)		0	(0)	
	Low	22	(57·9)	*·46*	11	(29·0)	*·15*	16	(42·1)	*·23*	5	(13·2)	***·06***
Direct/indirect involvement of skilled attendant: ^b^	Yes	10	(66·7)		1	(11·1)		4	(40·0)		0	(0)	
	No	33	(61·1)	*·77*	14	(23·3)	*·67*	30	(50·9)	*·73*	5	(8·5)	*·99*
Client’s parity status:	Nulliparous	15	(65·2)		2	(8·7)		8	(34·8)		3	(13·0)	
	Multiparous	28	(60·9)	*·80*	13	(28·3)	***·07***	26	(56·5)	*·13*	2	(4·4)	*·32*

Total *n* = 69 cases, numbers and frequencies shown represent only cases with observed care for each domain and characteristic

^a^ cutoff defined at 183 delivery cases in previous calendar year (i.e. on average one case every other day)

^b^ “skilled” includes nurses, midwives or other maternal care providers with any of the following training qualifications: A0 = 2-year postgraduate training, A1 = 3-year undergraduate training, A2 = 2-year undergraduate training; direct involvement = skilled provide directly performs observed care domain, indirect involvement = skilled provider supervises non-skilled provider in executing the observed care domain

*p*-values based on Fisher’s exact test

## Discussion

High quality intrapartum care is critical to the survival of mother and newborn. In this study, we assessed the quality of intrapartum care provided in two provinces of the DRC. Applying the “competent care and systems” component of a recently developed health systems framework allowed us to focus our quality assessment on selected key standards of intrapartum care provision.

### Competent care

Especially in rural settings of Sub-Saharan Africa routine partograph use in monitoring labor progression is often limited [25, 26]. This was also the cased in our study, where partograph-based monitoring was still absent in 15 cases albeit the availability of this tool. Partographs are commonly not or incorrectly used in instances where providers are either unfamiliar with its correct use as a clinical monitoring instrument [25, 26] or in instances where a high delivery caseload prevents diligent partograph documentation [27]. Since case volumes have on average been rather low across facilities in our study, inadequate partograph use in some of the observed cases therefore likely resulted from gaps in provider knowledge or effort with respect partograph-based labor monitoring.

In Cameroon partograph use was found more common in health clinics compared to hospitals [28]. While we did not find a relationship with respect to facility type, our study observed partograph use more commonly in nulliparous cases. Ideally there should be no preference in partograph use with respect to a client’s parity, but context-specific factors might influence decision-making processes in everyday clinical practice. For instance, in the DRC, nulliparous women face a higher risk of prolonged labor, fetal distress, and related negative birth outcomes [24, 29]. Provider attempts to using resources, such as partograph forms, more effectively might explain their more frequent use in higher risk patients.

With uterotonic drugs being a standard practice in routine management of stage 3 labor in Sub-Saharan Africa, absence of medical hemorrhage control usually results from stock-outs of the respective drugs [11, 30]. This is supported by our findings where oxytocin was administered in over 90% of cases. The five cases with no administration, oxytocin was actually in stock. However, these cases all occurred in facilities with lower case volumes, and omission of medical stage 3 management therefore might reflect providers’ deviation from standard practices as a result of irregular exposure to delivery cases and consequently lower familiarity with clinical standards. This seems to be further supported in our data by the fact that skilled providers were more likely to administer a uterotonic drug stage 3 labor management.

Clinical care during the immediate postpartum mainly consists of close patient monitoring to detect signs of birth-related complications (postpartum hemorrhage or neonatal asphyxia). However, only 41% of women delivering in a health facility in low- and middle-income countries receive postpartum health checks within the first hour following birth [15]. In 2014, only 27% mothers and 7% newborns in Kwilu and Kwango provinces received immediate postpartum checks within 4 hours after birth [8]. Similarly, our results indicate an almost abrupt discontinuation once care transitions from birth to postpartum. An explanation for this sudden drop in clinical monitoring activities might be existing misalignments in care processes, such as early transfer of woman and newborn from the labor room to other parts of the maternity ward or facility. Unfortunately, the data available to us did not allow a more detailed investigation to fully explain this finding.

### Deficient care

Our findings indicate that higher levels of infection prevention could have been provided to 45 of observed cases as functional handwashing facilities and sterile gloves were available at the respective facilities. In low- and middle-income countries, healthcare-associated infections related to facility-based deliveries are the leading cause of neonatal infections within the first 72 h of life [31]. In Western Africa handwashing prior patient exams occurred only in 6–11% of cases, the use of sterile gloves during vaginal exams in 5–21% [32]. Further, deficient infection prevention in our study was substantially more common in hospitals. This might have been a result of the often more variable composition of maternity teams in hospitals compared to health centers, as demonstrated by existing evidence on differences in hygiene precautions across different maternal care cadres [33].

Data from Sub-Saharan Africa suggests that delays in active stage 2 assistance commonly occurs in instances where birth attendants miss labor progression from first to second stage (e.g. due to delayed admission to labor ward, inadequate labor monitoring, understaffing) [34]. To capture measures preventing delays in care, we focused on the immediate accessibility to both routine delivery equipment and neonatal airway aspiration at stage 2 onset. Immediate accessibility, especially with respect to aspiration access, was rather deficient in our study. An earlier assessment of facilities in Kwilu and Kwango reported functional newborn suction devices available in only 18% of facilities [11]. This makes timely access not only the domain most affected by lack of equipment, but also demonstrates that non-functional equipment might be a rather persistent challenge in our study context.

### General considerations

Evidence suggests that health facilities with low volumes of institutional deliveries frequently fail to provide adequate clinical capacity (e.g. qualified maternal care staff, equipment) to ensure safe deliveries [22]. Especially facilities with case volumes below 500 births per year are more likely to produce low quality intrapartum care [22]. In our sample, all surveyed facilities fell under this threshold volume in the previous year. Furthermore, hospitals in our study had on average even lower volumes compared to health centers. While we did not observe any obvious relationships between lower delivery volumes and deficient care (except for the statistically significant association with prevention during stage 3), we did observe positive associations between competent care across most domains (for timely access during stage 2 even statistically significant) and higher volume facilities. The overall low case volumes across facilities in our study gives rise to the question whether alternative approaches to facility-based childbirth that produce higher case volumes across fewer facilities could be a potential path towards intrapartum care improvement in this rural context [35].

While adherence to evidence-based clinical standards is a common challenge in many countries in the region, data show only weak correlations between evidence-based care processes and the availability of essential care inputs [36]. This suggests that low quality of clinical care is predominantly a result of health provider’s unfamiliarity with clinical standards or other discrepancies between clinician’s knowledge and actual care provision (i.e. know-do gap) [37]. While our study was able to identify areas of deficient intrapartum care, it was unfortunately not conceptualized to further explore existing know-do gaps. Still, and particularly with respect to the deficiencies in patient safety and timely access, further research would be essential to identify and address such gaps.

### Limitations

Our study has several limitations. First, in combining information from different data collection streams, deficient care estimation might not have fully reflected actual availability of supply items (i.e. oxytocin, gloves, partograph forms) at the day of case observation, and therefore might have overestimated deficient care in instances where incomplete care was in reality a result of non-availability of respective supply items. Second, due to the Hawthorne effect, the degree of clinical adherence observed might be an overestimation of the quality of care provided in the absence of an observer, which would have mostly affected input-independent care processes (such as the continuity of care domain) or care where process-relevant inputs have actually been available. Lastly, while our facility sample represented a random sample of facilities enrolled under the PDSS program in each health zone, our findings might not be generalized to non-PDSS facilities within or any facility outside these zones. Similarly, as some sampled facilities had to be excluded from analysis due to missing case observations, this might have further limited the representativeness of findings with respect to the initially targeted sample.

## Conclusions

In the DRC, health facilities are responsible for implementing the national standards related to maternal, newborn and child health. However, facilities and providers in rural and remote areas face additional challenges doing so. Our study therefore tried to identify and assess shortcomings in intrapartum care quality in five health zones of two rural provinces.

Major gaps in competent intrapartum care included the provision of adequate infection prevention during childbirth, continuous clinical monitoring following delivery, as well as ensuring immediate access to essential obstetric equipment during childbirth. Each of these provision gaps can result in negative health outcomes for both mother and newborn and were more commonly observed facilities with overall lower delivery volumes. In addition, our findings suggest that deficiencies in care are largely a result of provider oversight and not necessarily a result of absent or non-functional equipment and supplies.

For health facilities to better address these gaps in intrapartum care, identified priority areas for health service management and quality improvement efforts should probably focus on staffing patterns and provider team compositions that allow better on-the-job supervision to ensure high level midwifery care can be provided at any time. In addition, there is a need to identify interventions that would strengthen protocol compliance, even by skilled birth attendants.

## Data Availability

The datasets used and/or analysed during the current study available from the corresponding author on reasonable request. Data used in this study will be made available upon request to investigators whose proposed use of the data has been approved by a review committee identified for this purpose.

## Abbreviations

DRC: Democratic Republic of the Congo
SBA: skilled birth attendant
